# FDA-Approved Oximes and Their Significance in Medicinal Chemistry

**DOI:** 10.3390/ph15010066

**Published:** 2022-01-04

**Authors:** Jyothi Dhuguru, Eugene Zviagin, Rachid Skouta

**Affiliations:** 1 Mitchell Cancer Institute, University of South Alabama, 1660 SpringHill Avenue, Mobile, AL 36604, USA; jyothi.dhuguru@gmail.com; 2 Department of Chemistry, University of Michigan, 930 N. University Avenue, Ann Arbor, MI 48109, USA; ezviagin@umich.edu; 3 Department of Biology, University of Massachusetts, Amherst, MA 01003, USA

**Keywords:** oximes, organophosphates, acetylcholinesterase, antidotes, pralidoxime, obidoxime, HI-6, trimedoxime, methoxime, cefuroxime, ceftizoxime, cefpodoxime, cefmenoxime, antibiotics, cephalosporins

## Abstract

Despite the scientific advancements, organophosphate (OP) poisoning continues to be a major threat to humans, accounting for nearly one million poisoning cases every year leading to at least 20,000 deaths worldwide. Oximes represent the most important class in medicinal chemistry, renowned for their widespread applications as OP antidotes, drugs and intermediates for the synthesis of several pharmacological derivatives. Common oxime based reactivators or nerve antidotes include pralidoxime, obidoxime, HI-6, trimedoxime and methoxime, among which pralidoxime is the only FDA-approved drug. Cephalosporins are β-lactam based antibiotics and serve as widely acclaimed tools in fighting bacterial infections. Oxime based cephalosporins have emerged as an important class of drugs with improved efficacy and a broad spectrum of anti-microbial activity against Gram-positive and Gram-negative pathogens. Among the several oxime based derivatives, cefuroxime, ceftizoxime, cefpodoxime and cefmenoxime are the FDA approved oxime-based antibiotics. Given the pharmacological significance of oximes, in the present paper, we put together all the FDA-approved oximes and discuss their mechanism of action, pharmacokinetics and synthesis.

## 1. Introduction

Oximes are the most common and widely acclaimed nitrogen containing biological motifs, with diverse biological and pharmacological applications. These hydroxy-imine derivatives are regarded for their antibacterial, anti-fungal, anti-inflammatory, anti-oxidant and anti-cancer activities [1,2]. Oximes have gained wide popularity due to their potency to act as antidotes against nerve agents. This is achieved by their ability to reactivate the enzyme—acetyl-cholinesterase (AChE) [3]. Organophosphates (OPs) are the compounds commonly used as pesticides, insecticides, medications and as nerve agents in chemical weapons. These compounds are extremely toxic and OP poisoning symptoms can vary from nausea, vomiting, diarrhea, muscle tremors and confusion, and can lead to fatality in a few minutes to days, depending on the toxicity of the agent [4]. Although the first OP insecticide was engineered in the mid-1800s, it only came to light after World War II.

**Figure 1:**
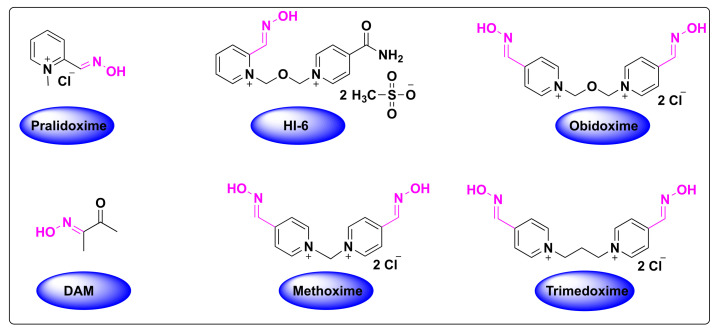
Chemical structures of oxime-based nerve antidotes.

A seminal work by Wilson et al., demonstrated that cholinesterase inhibition by the OPs can be reversed by hydroxamic acid and other oxime derivatives, and can therefore be used as powerful antidotes [5]. This discovery was further extended in the year 1958 by Grob et al., who reported two novel oximes named pyridine-2-aldoxime or pralidoxime (2-PAM) and diacetyl monoxime (DAM) as adjunts to atropine, which can reverse the neuromuscular block due to OP poisoning and activation of AChE [6]. Ever since the discovery of 2-PAM for organophosphate poisoning, a large number of bioactive compounds bearing oxime moieties were synthesized by various research groups towards the development of more effective antidotes. In addition to 2-PAM, other oxime derivatives, such as obidoxime, HI-6, trimedoxime and methoxime, were used as one of the standard antidotes for OP poisoning (Figure 1). Nevertheless, the antidote 2-PAM is the only FDA-approved drug until now, for the treatment of OP poisoning (Figure 1).

**Figure 2:**
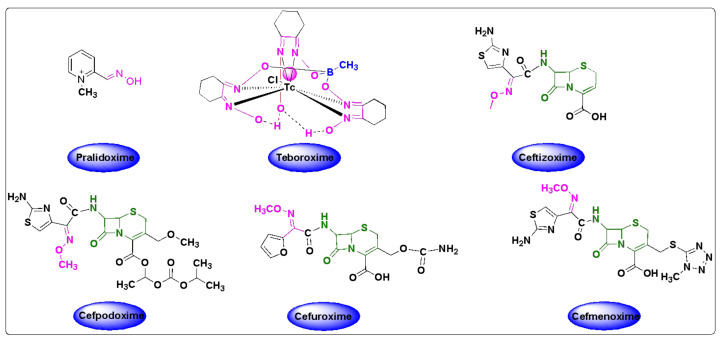
Chemical structures of FDA-approved oximes.

In addition to being used as OP antidotes, oxime-based cephalosporins are utilized as anti-microbial agents [7]. Among the β-lactam antibiotics, penicillin and cephalosporin drugs are the most widely and commonly prescribed anti-bacterial agents against infections [8]. The discovery of cephalosporin C in the 1950s, paved the way to the development of hundreds of novel cephalosporins [9]. Mechanistically, cephalosporin C is subjected to chemical or enzymatic hydrolysis to obtain 7-aminocephalosporanic acid, which serves as a key intermediate to synthesize several cephalosporin derivatives [10]. Oxime based cephalosporins are widely used drugs against bacterial infections and, among them, cefuroxime, ceftizoxime, cefpodoxime and cefmenoxime are FDA-approved oximes used against several bacterial infections (Figure 2). Their synthesis and pharmacokinetics will be discussed in this review.

### 1.1. The Sources and Discovery of Oximes

Oximes constitute a very important class of compounds in plants and serve as key players in the metabolism and a variety of biosynthetic pathways [11,12]. A wide range of oximes are found in plants and several structurally diverse oximes are involved in the metabolism of plant growth and development. The most common varieties include volatile organic compounds, cyanogenic glucosides and glucosinolates, which explains the ubiquitous nature of these enzymes in plants [11].

Early reports on the occurrence of oximes in plants were reported by Tapper et al., in 1967 [13]. They identified the isobutyraldoxime compound from linen flax (*Linum usitatissimum*), during their investigation on cyanoglucoside linamarin [13]. The cyanoglucoside compounds family are most commonly encountered in plants, and play a vital role in the plant’s defense mechanism by the release of hydrogen cyanide. Concurrently, preliminary reports on their occurrence in the biosynthesis of glucosinolates were presented by Underhill et al., who isolated and identified phenylacetaldehyde oxime via phenylalanine from *Tropaeolum majus* shoots through their C14 labeling experiments [14]. Phenylacetaldehyde oxime was found to be the precursor of the glucosinolate glucotropaeolin. In members of *Brassicaceae* and *Arabidopsis*, the formation of indole-3-acetadoxime from tryptophan serves as an important biosynthetic pathway for the biotransformation to other important metabolites, such as camalexin, via indole-3-acetonitrile, auxins, such as indole-3-acetic acid, and other indole-3-glucosinolates [15,16]. An extensive review by Mahadevan et al., in 1973, described the role of oximes as intermediates in their biotransformation to amino acids and secondary metabolites, such as cyanogenic glucosides, nitriles and other derivatives [17]. Notably, oximes in plants are derived from amino acids through biosynthetic pathways catalyzed by the CYP79 family—a class of enzymes that are known to play a critical role in both normal and specialized metabolism [11].

### 1.2. The Synthesis of Oximes

Oximes have gained wide interest in the field of organic chemistry due to their ease of synthesis from the carbonyl compounds, and their facile conversion to nitriles, amines, nitro compounds and other heterocyclic compounds [18]. In addition, they are popular as protecting groups of carbonyl compounds and as intermediates in the Beckmann rearrangement for the synthesis of important β-lactam derivatives [19]. Oximes have wide ranging industrial applications and a classic example of their significance is indicated by the great production of caprolactam, which is a precursor to nylon-6, whose production exceeds over five million tons per year [20].

The classical method of oxime synthesis involves the treatment of hydroxylamine with aldehydes or ketones. Oximes can also be derived from non-carbonyl compounds, and the reduction of nitroalkenes is the most useful method to generate both aldoximes and ketoximes [21]. The reduction of α,β-unsaturated nitroalkenes (when the nitro group is terminal) with a slightly acidic medium, such as tin (II) chloride, results in aldoximes in a high yield. On the other hand, the reduction of α,β-unsaturated nitroalkenes (when the nitro group is internal) with alkaline medium, such as sodium stannite (Na_2_SnO_2_), yields ketoximes in high yields [22]. In addition to oximes, oxime ethers and esters, amidoximes have also gained the attention of organic chemists in the recent years, owing to their ability to generate a variety of heterocyclic compounds that are of great biological and pharmacological interest. The detailed description of the synthesis of oximes and their derivatives were extensively reviewed [22,23].

### 1.3. Isomerism of Oximes

Oximes generally exist in the form of interconvertible *E* and *Z* stereoisomers, depending on the orientation of groups around the C=N bond [24]. These isomers can have different physical properties and can be readily isolated by chromatography or recrystallization techniques. Oxime stereoisomers have important pharmacological properties and studies revealed that *Z*-isomers are more stable and predominant than *E*-isomers. Differential scanning calorimetry studies also proved this fact that heating and melting of *E*-oxime readily afforded the *Z*-oxime. Interestingly, the reagents used for the synthesis of oximes from aldehydes and ketones can also catalyze the interconversion of *E* and *Z* isomers. Temperature plays a critical role in the determination of the isomer ratio, as any change in the temperature can vary the position of equilibrium and subsequently alter the equilibration ratio of the isomer mixture [25].

The discussion on oxime isomerism cannot be complete without referring to other tautomers of oximes [26]. Three main tautomeric forms have been identified, namely oximes, nitrones and nitroso compounds. Oxime-to-nitrone isomerization through a thermal 1,2-hydrogen shift was first identified by Grigg et al. [27] Both nitrone and nitroso tautomers were found to be less stable than oximes. Nitrones are found to be more reactive than oximes, and, therefore cannot be isolated, but can be trapped as intramolecular cycloaddition products [28].

### 1.4. Bioisosterism and Its Pharmacological Relevance in Oximes

**Figure 3:**
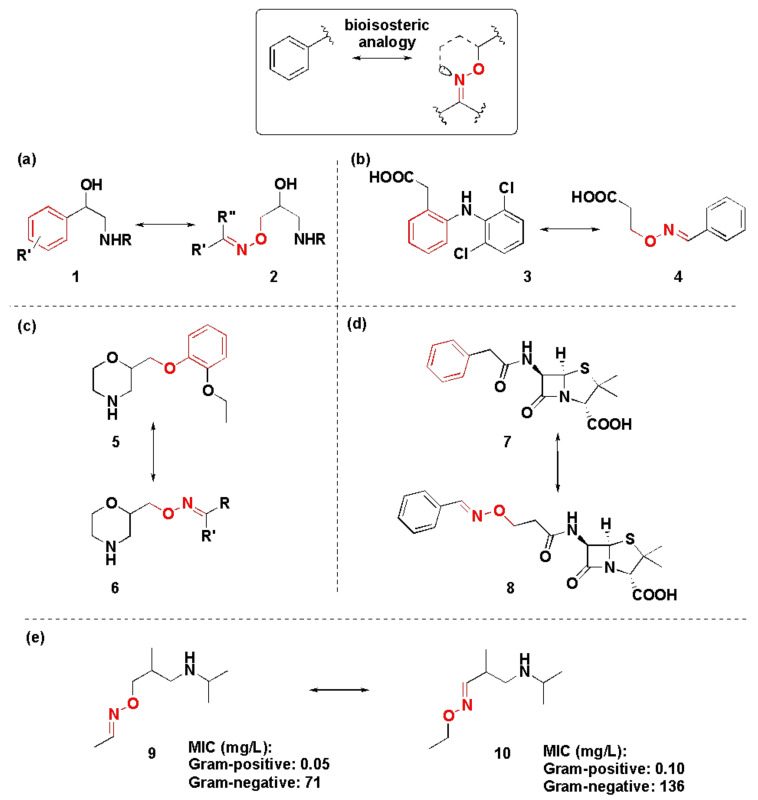
(**a**–**e**): Representative structures of MAOM moiety-modified bioisosteres of common bioactive compounds.

Jung et al. proposed 1,2,3-triazole as an isostere for oxime in the synthesis of potential cytochrome P450 stabilizers [29]. On the other hand, Patani and LaVoie reported methyleneaminoxy methyl (MAOM) moiety as a bioisostere for benzene rings (Figure 3a) [30]. In this way, the oxime ether **2** can serve as a β-adrenoblocker (Figure 3a), and an anti-inflammatory agent **4** analogous to diclofenac **3** (Figure 3b). Morpholine-based antidepressants, similar to viloxazine **5**, modified with MAOM (**6**, Figure 3c) showed similar in vivo and in vitro results. The same bioisosterism principle was applied for the modification of penicillins; however, the minimum inhibitory concentrations were significantly increased when using derivative **8** instead of penicillin G **7** (Figure 3d). The inversion of the oxyimino group also proved to be isosteric in terms of pharmacophoric homology and chemical reactivity, as in the case of β-adrenergic activity for derivatives **9** and **10** (Figure 3e). Oximes also showed comparable results to ketones, in the studies of LTB4 inhibition. However, most importantly, oximes and oxime ethers can be used as analogs for phosphate groups and for peptide bonds [31,32,33]. Potentially, such bioisosterism can lead to the discoveries of new therapeutic agents for the treatment of nucleotide and protein-related illnesses.

**Figure 4:**
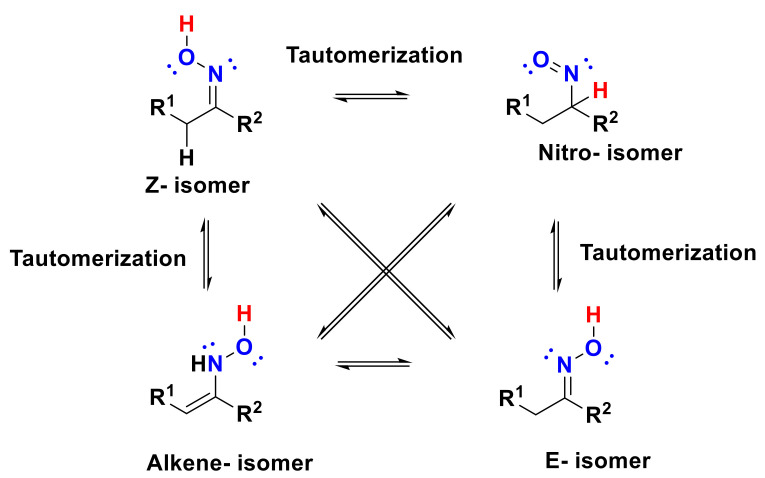
Isomerism in oximes.

The advantages of using oximes-based derivatives as therapeutic agents can be explained by a close investigation of the structure of the hydroxyimino (or alkoxyimino) group (Figure 4) [34]. Due to tautomerism and the possibility of *E*- and *Z*-isomers of oximes, this moiety proves its universality and can thus be used for various purposes of medicinal chemistry. This is evident by the tautomerization observed in the formation of the nitro isomer, as shown in Figure 4. Another reason of oxime relevance for the pharmaceutical industry is its ability to play a dual role as a hydrogen bond donor and an acceptor.

## 2. Classification of FDA-Approved Oximes

Despite the growing number of oxime-based compounds that have passed clinical testing and are already widely used for medicinal purposes, the FDA approved only six of them, which include pralidoxime, teboroxime (a myocardial imaging agent) and four oxime-based cephalosporines. We summarize the known pharmacokinetic properties of these FDA-approved oximes, the state-of-the-art acknowledged mechanism of their action, their close analogs and synthesis. As teboroxime is an imaging agent and does not have a therapeutic activity, in this short review, our center of focus will be on OP treatment agents and oxime-based cephalosporines. The FDA-approved oxime-based drugs can be classified into two categories, namely (a) oximes as organophosphate poisoning antidotes and (b) oximes based cephalosporin antibiotics.

### 2.1. Oximes as Organophosphate (OP) Poisoning Antidotes

Tetraethylpyrophosphate (TEPP) was the first OP, synthesized in 1854 by the French chemist Philippe de Clermont [35]. The general structure of OPs was first provided by Schrader in 1937, which is comprised of esters or thioesters or anhydride derivatives of phosphoric acid [36]. OPs contain a facile leaving group, which is prone to hydrolysis and is eliminated by the phosphorylation of acetylcholinesterase [37].

Before the administration of oximes for OP poisoning, it is critical to remove any OP residues from the patient. Depending on the affected area of poisoning, which can be either the skin (dermal), eyes (ocular) or by ingestion (gastric), decontamination is the preliminary approach to relieve the patient of OP poisoning [38,39]. While the exposed regions must be thoroughly decontaminated using soap and water, oral ingestion entails the need of gastric aspiration and lavage [38]. Decontamination methods also encompass the use of hypochlorite, Fuller’s earth, sodium hydroxide or sodium bicarbonate, depending on the scale of OP poisoning. In addition to the use of antidotes, other physical and photochemical methods are also being applied for decontamination. Photochemical methods, such as photolysis, photocatalysis and photosensitizers, and the use of metal organic frameworks, non-thermal plasma, cyclodextrins and gamma irradiation are among other common methods for OP decontamination [40,41,42,43]. After the preliminary steps to counteract the OP poisoning, a second strategy will be to administer bioscavengers, either through aerosols or intravenously to purge the patient from the toxins. These bioscavengers are of three types, which include stoichiometric, catalytic and pseudocatalytic classes [44]. The stoichiometric category includes the single-use enzymes, while the catalytic group has a high turnover and can be divided into either mammalian and bacterial types, based on the source of the enzyme. Some bacterial enzymes include phosphotriesterase, methyl parathion hydrolase and organophosphate acid anhydrolase, while paraoxonase, the human senescence marker protein and human triphosphate nucleotidohydrolase are some of the mammalian catalytic bioscavengers employed for the OP poisoning treatment [44,45]. The treatment of OP poisoning by using oximes, which is our current discussion, and other enzyme inactivators falls under the pseudocatalytic type [39].

**Figure 5:**
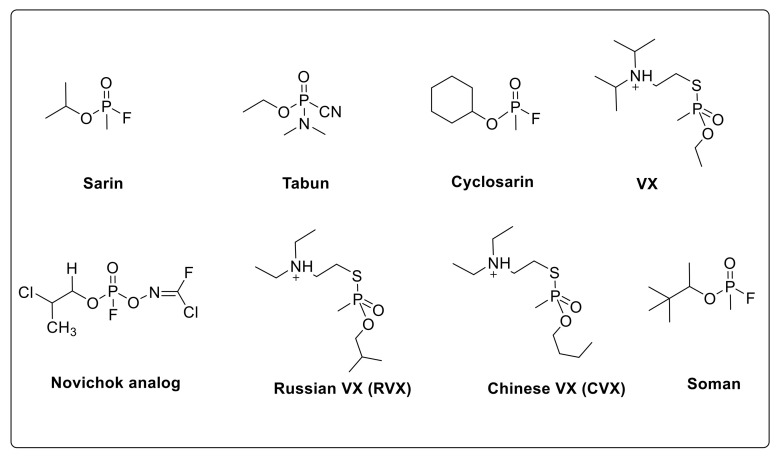
Chemical structures of selected organophosphate agents.

The most important application of oximes is their ability to treat OP poisoning by the reactivation of acetylcholinesterase, which is inactivated by the OPs that form the dimethyl– or diethylphosphoryl–acetylcholinesterase complex [24,46]. The current known organophosphates that are used as nerve poisons include sarin, cyclosarin, tabun, VX, Russian VX, Chinese VX, soman and novichok (Figure 5) [47,48,49,50]. A detailed investigation on the OP poisoning and the development of AChE reactivators as antidotes appeared in the literature [51]. A recent literature review by Worek et al., focused on the area of OP poisoning and lists the systematic development of oxime reactivators to counter the toxic effects of OP derivatives [52].

### 2.2. Significance of Acetylcholinesterase and Its Inhibition in OP Poisoning

Acetylcholinesterase (AChE, also known as acetylcholinehydrolase) is an enzyme that catalyzes the hydrolysis of neurotransmitter acetylcholine to choline and acetic acid (or acetate) [53]. Acetylcholine is a major neurotransmitter found in the central and peripheral nervous systems, best known for the transmission at the neuromuscular junction and at ganglionic synapses, and is broken down by the action of AChE [36]. AChE is composed of a serine site and an anionic site. The serine site is embedded in the active site of the enzyme and is prone to OP attack, ultimately resulting in the phosphorylation of serine site and the inactivation of AChE through the formation of a strong covalent bond. This irreversible binding results in the AChE inhibition by excessive cholinergic stimulation on the nicotinic and muscarinic receptors. This overstimulation can result in a variety of symptoms based on the type of receptor. Hyperstimulation of muscarinic receptors can lead to nausea, vomiting, diarrhea, seizures, abdominal cramps, incontinence of feces and urine, while the hyperactivation of nicotinic receptors can result in hypertension, muscle cramps, fasciculations, tachycardia and paralysis [54]. Death in patients due to OP poisoning, commonly occurs from the respiratory failure. 

**Figure 6:**
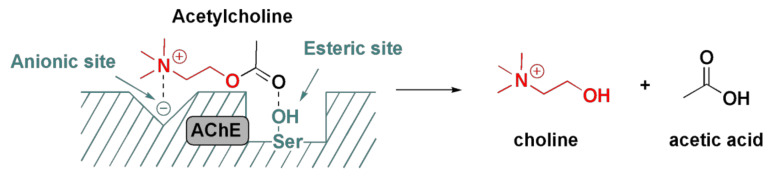
Mechanism of action: role of the acetylcholinesterase (AChE) enzyme in the hydrolysis of acetylcholine.

The normal mechanism of action of AChE (Figure 6) includes the binding of acetylcholine to the anionic site via its positively charged quaternary nitrogen, and to the serine hydroxyl group via a hydrogen bond with the carbonyl oxygen of acetyl group [34]. Such combined action fixates acetylcholine in a position that strongly favors a nucleophilic attack by the water molecule on the carbonyl group, and, hence, the hydrolysis of the molecule, as shown in Figure 6. 

### 2.3. Detoxification of OP Poisoning by Pralidoxime

**Figure 7:**
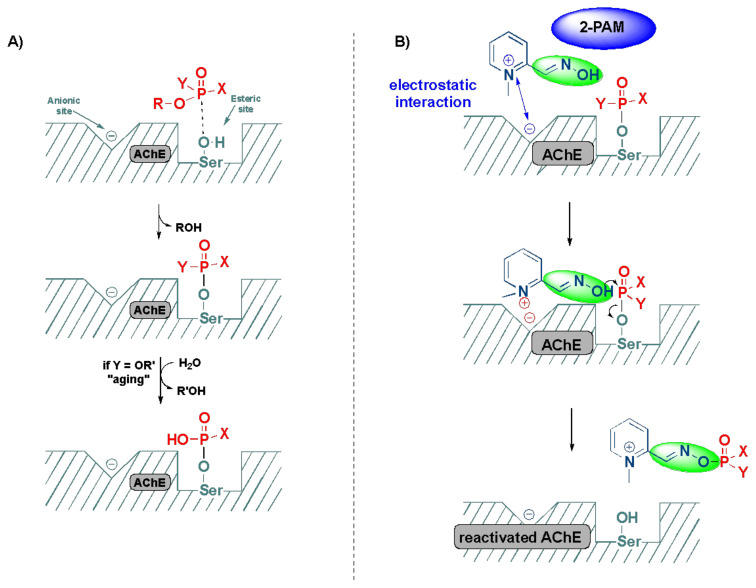
(**A**) Organophosphate poisoning of AChE. (**B**) Reactivation of AChE by pralidoxime.

The inactivation of AChE by OPs leads to the accumulation of acetylcholine in the exposed patients, resulting in the overactivation of muscarinic and nicotinic receptors, which ultimately culminates in the range of aforementioned symptoms [55]. The mechanism of action of the reactivation of AChE by oximes can be best described using the FDA-approved drug, 2-pyridine-aldoxime methyl chloride (2-PAM) (also known as pralidoxime), as a classic example (Figure 7). The 2-PAM is the most popular and well-investigated agent, along with HI-6 and obidoxime [4,36,56]. The 2-PAM has a positively charged quaternary nitrogen atom of the pyridinium ion that specifically interacts with the anionic site of the AChE, and, then, the hydroxyl group of the oxime moiety replaces the serine residue by nucleophilic substitution, releasing the free hydroxy (OH)-group of the amino acid (Figure 7) [57,58,59]. This can be explained due to the higher affinity of 2-PAM to become phosphorylated over the serine residues by the OP agent, and thereby liberating the enzyme and making it available to bind to acetylcholine [53]. This AChE restoration relieves the associated symptoms of OP poisoning, such as muscle twitching and tremors, convulsions, difficulty breathing and fatigue [36].

The major limitation of oxime treatment in OP poisoning, lies in the fact that none of these drugs can effectively reactivate the AChE inactivation induced by all OP compounds, and their efficacy is dictated by the therapeutic window, controlled by the time of administration and “aging” [60,61]. As observed earlier, OP compounds have a facile leaving group that is lost during the serine phosphorylation; if an additional alkyl group is lost in the process producing an oxyanion on the phosphoryl group, then this process is referred to as EagingE. Aging in OPs stabilizes the oxyanion with the positively charged histidine residues by the electrostatic attraction, thereby making the AChE resistant to the reactivation by oximes [61].

## 3. Computational and SAR Studies of AChE Reactivators

Several studies based on molecular modeling and computer simulations appeared in the literature, to aid the experimental research in the efforts to design efficient oximes for the deactivation of a broad spectrum of OP compounds, as no single oxime available in the market can target the broad spectrum of OP derivatives [62,63,64]. For instance, docking studies and DFT calculations, conducted by Ramalho et al., demonstrated that the theoretical data aligned well with the experimental results, in the prediction of free energies of oximes that can further aid in designing effective oximes targeting AChE [65].

Jokanovic and Prostran reviewed the known structure–activity relationship (SAR) studies of oxime-based antidotes, including 2-PAM [66]. Their key findings revealed that (a) the presence of quaternary cationic nitrogen is essential; (b) the length of the linker between the two bispyridinium rings affects their potency; and (c) better results were observed for compounds in which the hydroxyimino group is in position **2** or **4** in the pyridinium ring relative to the nitrogen atom, due to the hydroxyimino increased acidity. 

**Figure 8:**
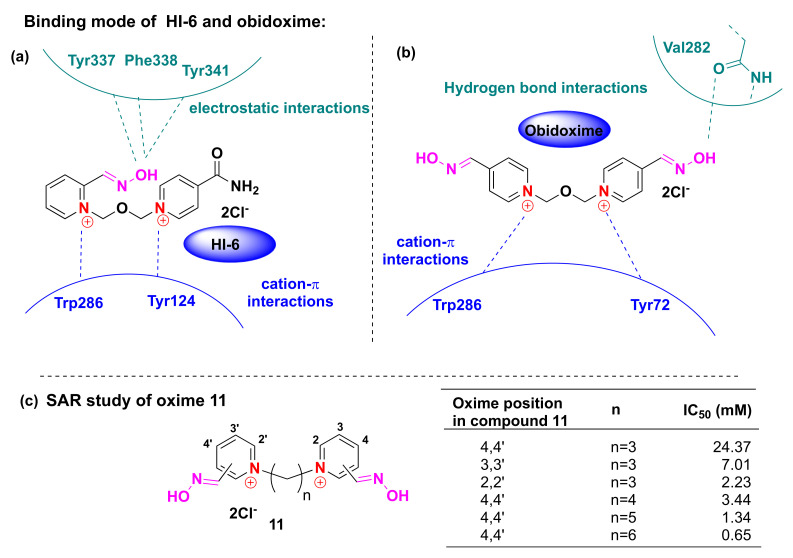
Binding modes of (**a**) HI-6, (**b**) obidoxime and (**c**) structure activity relationship (SAR) study based on oxime **11** dependence of IC_50_ (half maximal inhibitory concentration) for the reactivators of the human recombinant AChE, PDB# 2GYW (stated are the positions of oxime groups in the pyridinium rings related to the nitrogen atom, number of carbon atoms in the linker and IC_50_ of the reactivation of acetylcholinesterase using compounds **11**).

The in silico docking study of oxime-based OP antidotes such as HI-6 and obidoxime against the 3D crystal structure of mouse AChE (Figure 8: PDB: 2GYW) was reported by Bhattacharjee and co-workers [34]. Their studies showed that binding to the potential active site of AChE occurs via strong cation-π interactions between the Trp286 and Tyr124 side chains, and the 4-carboxylamine-pyridinium ring of the HI-6. Tyr337, Phe338 and Tyr341 were also reported to interact via nonbonding contacts and hydrogen bonds to the 2-hydroxy-iminomethylpyridinium ring of HI-6. Additional analysis of the binding mode, showed the hydrogen bond donor of the oxime group with Val282 and via cation-π interactions with Trp286 and Tyr72 side chains. The hydroxyl group of the oxime was also found to form hydrogen bonding acceptors with the Phe 295 and Arg 296 side chains. Authors also stated that the binding of obidoxime to AChE, proceeds through the cation–π and π-π interactions of both pyridinium rings with the sidechains of the AChE protein (Tyr124, Trp286, Tyr72, Tyr341 and Phe338). More importantly, oximes were showed to form a hydrogen bond with the carbonyl oxygen of Val282 (Figure 8). This highlights the significance of the oxime group in the OP antidotes [67].

Sepsova et al. investigated the SAR studies of twenty four various oximes, which are close structural analogs of 2-PAM and HI-6, and obtained by changing the position of the hydroxyimino group in the aromatic ring and the nature of the linker between two pyridine fragments [68]. The tests were carried out for human recombination AChE and the results were compared to the earlier reports. They confirmed that the highest activity is observed for the ortho-oximes, and the size of the linker should be larger than five carbon atoms (Figure 8).

Musilek et al. reviewed a variety of the different AChE reactivators discovered by that time and discussed their structure–activity relationship, including oxime- and non-oxime-based reactivators [69]. They concluded that mono-oxime based compounds performed better than bis-oximes, whereas bis-quaternary compounds are preferable over mono-quaternary derivatives. At the same time, the non-oxime part of the oxime reactivator is also very important, as it was stated for molecules containing carbamoyl, methylcarbonyl or isoquinolinium moieties. However, Gorecki et al. recently reported the efficacy of uncharged non-zwitterionic bis-oximes, showing their SAR by in silico experiments [70].

Castro and Figueroa-Villar performed calculations using Hartree–Fock, Møller–Plesset and density functional theory (DFT) methods, such as B3LYP, for the determination of rotational barriers, charge distribution and some geometrical and equilibrium parameters for both protonated and unprotonated (zwitterion) forms of pralidoxime [71]. These two forms are present in the physiological conditions and the mechanisms of action are still an object of debate. Despite the fact that the unprotonated form is biologically active, its rigidity due to conjugation and the weaker nucleophilicity of oxygen makes its hard to bind to the active center of acetylcholinesterase, thereby favoring the protonated form. This arrangement makes the side chain flexible, to adjust to the geometry of the enzyme. The weaker nucleophilicity of the oxygen results from the contribution of the non-aromatic resonance form that decreases the partial negative charge of atoms.

Silva et al. later investigated the geometric parameters and charge distributions of another organophosphate antidote—HI-6 [72]. The results for the charge densities for oxime oxygen were contradictory, and, therefore, could not be used to indicate the existence of resonance forms for the HI-6 molecule. The general trend for negative charge density of the side chain oxygen atom favors various electrostatic interactions, which can explain the high efficiency of HI-6 in the treatment of OP poisoning. Further studies on the rationalization of the structure of the AChE reactivator to lower the toxicity of the substance and increase its lipophilicity and reactivation rate are in progress [73,74].

Table 1 is a compilation of a short comparison of the most important pharmacokinetic parameters for oxime-based AChE reactivators that are already in use, and some most promising experimental oximes. Bohnert et al. also recently reviewed the pharmacokinetic data of 2-PAM, HI-6 and obidoxime, as parts of OP poisoning treatment (in combination with each other and atropine) [56]. The problem of slow development of this class of compounds is due to their slow transition from clinical trials to real-time applications, as it occurred to HI-6 in the past [52]. From the latest data, one of the most promising oxime reactivators is K203 [75].

**Figure 9:**
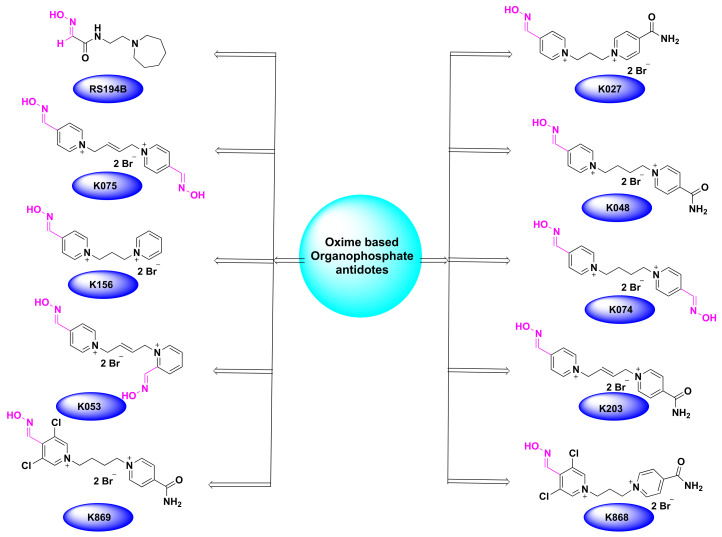
Chemical structures of oxime-based organophosphate antidotes.

The most recent data showed that several oximes were effectively used, even as pretreatment for the paraoxon poisoning [76]. Examples include RS194B, K027, K074, K075, K156, K868 and K869 (Figure 9). Although there have been significant advancements during recent years, there is a crucial need for the development of new oxime derivatives to overcome the shortcomings of the existing ones, such as the low lipophilicity of oxime-based compounds and improving the blood–brain barrier penetration [77,78]. In this direction, continuous research is in progress for the development of more efficient oxime derivatives to reduce the toxicity and increase potency [79].

## 4. Oxime Based FDA Approved Drugs

### 4.1. Pralidoxime

Pralidoxime is an FDA-approved acetylcholinesterase reactivator and is the most used oxime for the treatment of OP poisoning. In addition to being approved as an antidote for the nerve agent poisoning, it is also most often used as an antidote for OP poisoning in conjunction with atropine. Although it exists in various other salt forms, such as mesylate, methiodide and methyl sulfate forms, chloride is the most predominant form of 2-PAM, owing to its low molecular weight, excellent water solubility and high potency [53,54,80]. The minimum therapeutic amount of 2-PAM was reported to be 4 µg/mL in the blood plasma, which can be reached within 15 min of a single intramuscular injection, and a similar dose can be repeated after one hour of the symptoms [81]. Maximum concentration is reached within 35 min [53,80]. As noted earlier, pralidoxime acts by the binding of the pyridine moiety next to the damaged organophosphate-modified serine site in the AChE, and the nucleophilic attack of the phosphorus atom by the oxime oxygen.

Pralidoxime is an active ingredient in medications, such as Atnaa, Duodote (with atropine), pralidoxime chloride and protopam chloride (Table 2) [82].

**Scheme 1:**
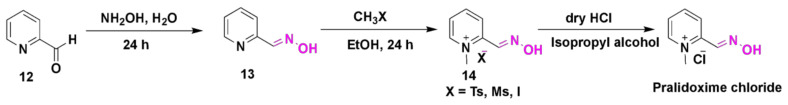
Synthetic scheme of pralidoxime chloride.

Pralidoxime chloride is synthesized, as shown in the synthetic Scheme 1 [83]. The first step involves the treatment of pyridine-2-carboxaldehyde (**12**) with hydroxylamine hydrochloride to obtain an aldoxime intermediate **13**. The resulting oxime (**13**) is further methylated using a corresponding methylation agent, to obtain N-methylated-pyridinium intermediate (**14**). The final step involves the treatment of compound **14** with dry HCl gas in isopropyl alcohol, to afford the desired 2-pralidoxime chloride.

**Table 2:** FDA-approved oximes, their brand names (discontinued and active) and medicinal applications. **: cited references.

Structure	Brand	Applications (References) **
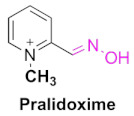	Atnaa, Duodote (with atropine), Pralidoxime Chloride, Protopam Chloride	Organophosphate poisoning and pre-treatment [57,82].
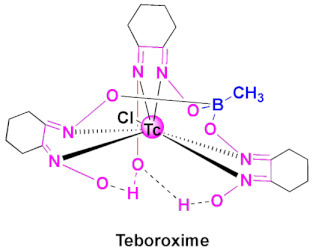	Cardiotec	Agent for myocardial perfusion imaging [84,85].
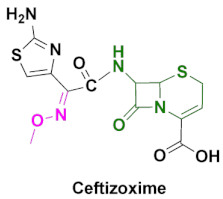	Cefizox	Gonorrhea, pelvic inflammatory disease, urinary tract infections, cystitis, epiglottitis, meningitis, osteomyelitis, pneumonia, skin/soft tissue infection and other diseases caused by Gram(+) and Gram(-) bacteria [86].
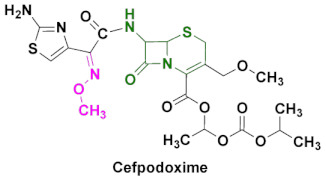	Banan, Vantin, Cefpodoxime Proxetil	Acute bronchitis, pneumonia, pharyngitis/tonsillitis, gonorrhea, urinary tract infections, otitis and other diseases caused by Gram(+) and Gram(-) bacteria [87,88,89].
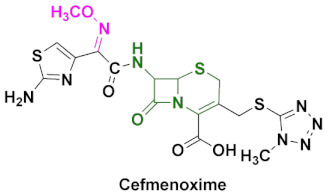	Cefmax	Treatment of female gynecologic and obstetric infection, gonorrhea, otitis, skin/soft tissue infection, sinusitis and other diseases caused by Gram(+) and Gram(-) bacteria [90,91].
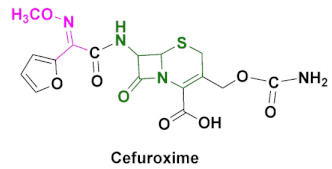	Ceftin, Cefuroxime, Cefuroxime sodium, Kefurox, Zinacef	Skin and middle ear infections, tonsillitis, throat infections, laryngitis, bronchitis, pneumonia, urinary tract infections, gonorrhea and other diseases caused by Gram(+) and Gram(-) bacteria [92,93].

### 4.2. Teboroxime

**Scheme 2:**
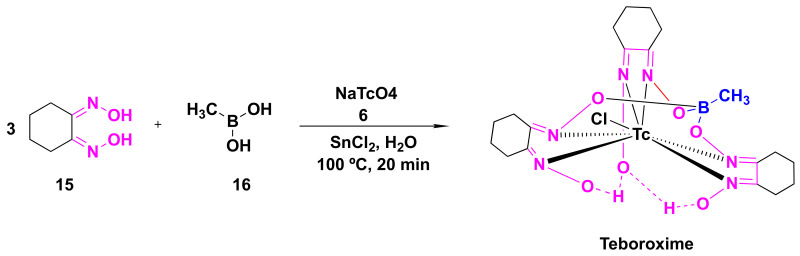
Synthetic scheme of teboroxime.

Teboroxime or technetium-99m-teboroxime (Tc-99m-teboroxime) is a small, neutral, lipophilic mixture obtained from the boronic acid adducts of technetium dioxime complexes (BATOs) [94,95,96]. It is synthesized from methylboronic acid, stannous chloride and 99m-technetium salts following the synthetic route, as shown in Scheme 2. It is used as a myocardial perfusion imaging agent, ever since FDA approval in the year 1991 [97,98]. Tc-99m-teboroxime was then withdrawn from the market because of rapid clearance kinetics that did not allow high qualities of the images [85]. Nevertheless, it was taken back to the market, in some countries, for the solid-state photon emission computed tomography (SPECT). Its pharmacokinetics and distribution were discussed in relation to coronary artery disease and ischemia [99,100]. Teboroxime was the active ingredient in previously FDA-approved Cardiotec (Table 2) [101].

### 4.3. Oxime-Based Cephalosporins

β-lactam antibiotics: The discovery of *Penicillium* by Alexander Fleming, in 1928, represents a remarkable milestone in the history of science, as it revolutionized the field of medicinal chemistry towards the development of various anti-bacterial drugs against a spectrum of bacterial infections [102,103]. Today, β-lactam antibiotics have emerged as the most common and widely used tools to treat the bacterial infections and include several classes, such as penicillins, cephalosporins, carbapenems, β-lactamase inhibitors and monolactams [8,104,105]. They inhibit the bacterial cell wall synthesis by interfering with the biosynthesis of peptidoglycan, which occurs when antibiotics bind to specific penicillin binding proteins involved in the transpeptidation reactions, ultimately leading to bacterial cell lysis and death [8,10]. Cephalosporins are a large group of β-lactam derivatives, first isolated from the fermentation products of *Cephalosporium acremonicum* near a sewage coast of Sardinia [9].

**Figure 10:**
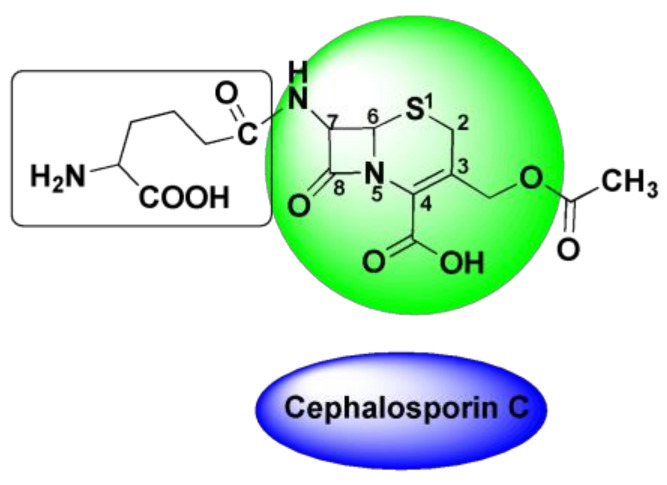
Structure of cephalosporin C.

Cephalosporins: Cephalosporins are comprised of a large amount of cephalosporin C, which is made of 7-amino-cephalosporanic acid (7-ACA) and alpha-aminoadipic acid [7,9,10]. As shown in Figure 10, cephalosporin core is comprised of a β-lactam ring tethered to a 6-membered dihydrothiazine ring. This structure sets them apart from penicillins and renders them resistant to the β-lactamase action of microbes. Cephalosporins can be derivatized into several types, based on two primary structural modifications-one on the amino group of the β-lactam ring and the other on the 3rd position of dihydrothiazine ring.

**Figure 11:**
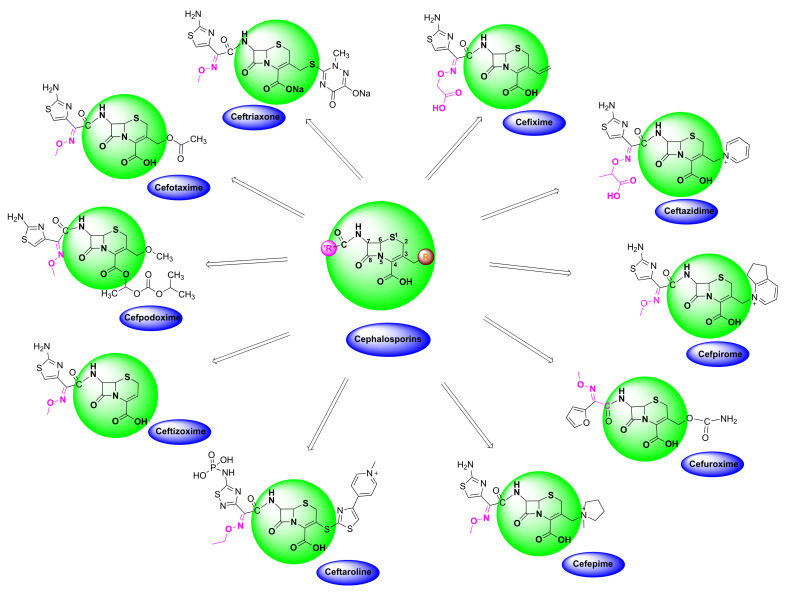
Representative examples of cephalosporin-based oximes.

Cephalosporins are categorized into five groups, usually designated as first to fifth generations, based upon their discovery and spectrum of anti-bacterial activity [10,104]. The most recent ones include fifth generation cephalosporins. First generation cephalosporins have a wide range of anti-bacterial activity against Gram-positive cocci and enterobacteria, but limited activity against Gram-negative microbes. Second generation cephalosporins are less potent against Gram-positive bacteria, but have improved activity against Gram-negative bacteria, compared to the first generation. The most popular oxime-based drugs in this class includes cefuroxime (Figure 11). Third generation cephalosporins exhibit extended activity against Gram-negative pathogens, and this class is known for their resistance to β-lactamase and shows a high potency against resistant strains of Gram-negative bacteria. 

Oxime-based cephalosporins of this class include ceftriaxone, ceftazidime, cefotaxime, ceftizoxime, cefixime and cefpodoxime (Figure 11). Fourth generation cephalosporins have a wide spectrum and improved activity against both Gram-positive and Gram-negative bacteria. Cefepim and cefpirome belong to this category (Figure 11). Fifth generation cephalosporins includes ceftaroline with a broad-spectrum anti-bacterial activity, and its ability to treat methicillin-resistant *Staphylococcus aureus* and other pathogenic strains, such as *Listeria monocytogenes* and *Enterococcus faecalis*, makes it unique among other classes.

### 4.4. Cefuroxime

**Figure 12:**
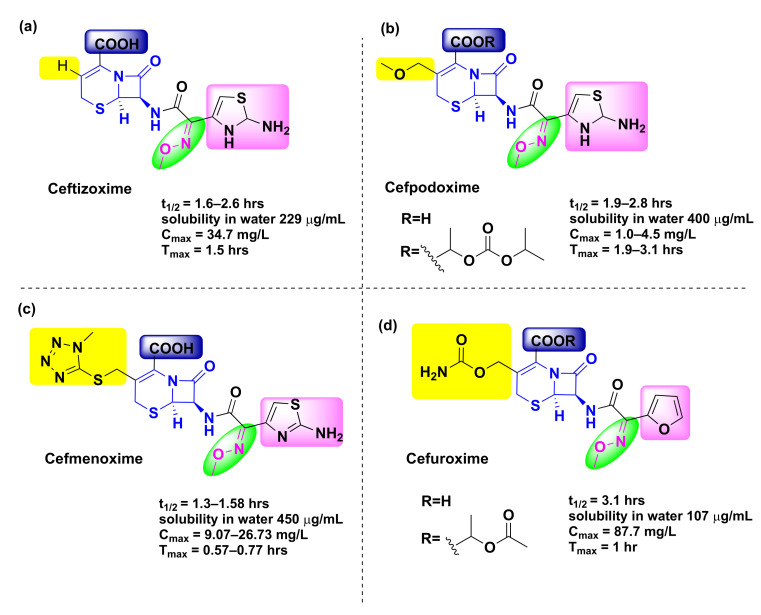
Pharmacokinetic parameters for (**a**) ceftizoxime, (**b**) cefpodoxime, (**c**) cefmenoxime, and (**d**) cefuroxime compounds.

Cefuroxime is a second generation cephalosporin, usually sold in the form of its Axetil ester (Table 2 and Figure 12d) [92,93,106]. It is reported that it takes less than 3 min to be converted to free acid, once the substance is in the body. Its bioavailability is considered to be higher in a fasting state than after a meal, which needs further validation. It is variably distributed in organs and tissues, but is shown to accumulate in the respiratory tract tissues. Most anti-bacterial medications of this class are shown to induce nausea, vomiting, diarrhea and gastrointestinal side-effects [92,107]. This semisynthetic antibiotic is reported to be effective against lower respiratory tract infections, skin and soft tissue infections and even on the early stages of the Lyme disease [108]. Its pharmacokinetics, therapeutic efficacy and pharmacodynamics are thoroughly reviewed in recent publications (Table 3). Cefuroxime is an active ingredient of medications, such as discontinued Ceftin, Cefuroxime, Cefuroxime sodium, Kefurox and currently prescribed Zinacef (Table 2) [109].

**Scheme 3:**
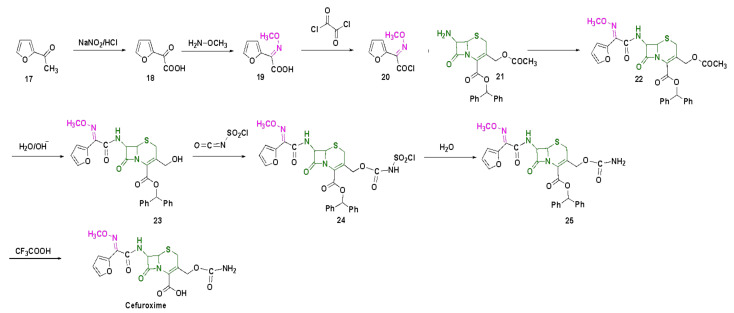
Synthetic scheme of cefuroxime.

The synthetic scheme of cefuroxime is shown in Scheme 3 [104]. The first step involves the treatment of 2-acetyl-furan (**17**) with nitrous acid to obtain compound **18**, which is further converted to a methoxy-imine intermediate (**19**), by the reaction with methoxylamine. Compound **19** is further treated with oxalyl chloride to obtain compound **20**, which is then converted to **22** by the reaction with a 7-amino-cephalosporanic acid intermediate (**21**). Compound **22** is deacetylated and converted to **24** via intermediate **23** using chlorosulfonyl isocyanate. Compound **24** is further hydrolyzed to **25**, and the final step of benzhydryl deprotection using trifluoroacetic acid, afforded the desired product of cefuroxime.

### 4.5. Ceftizoxime

Ceftizoxime is a third-generation cephalosporin anti-bacterial agent, introduced intravenously or intramuscularly (Figure 12a) [110,111]. Just like other third-generation antibiotics, it is active against many Gram-positive and Gram-negative bacteria, including those that have evolved to produce β-lactamases [110]. The main adverse effects of the drug include gastroenteric tract symptoms, such as nausea, vomiting, diarrhea and rashes [112]. The rate of binding to the protein is tested to be 30%, and t_1/2_ of the subject compound is proved to be around 1.5 h (Table 3). Additional details from the pharmacokinetic studies are presented in Table 3. The cumulative review informs us that ceftizoxime is particularly good for the treatment of lower respiratory tract and skin and soft issue infections [86]. Ceftizoxime is an active ingredient of discontinued Cefizox (Table 2) [111].

**Table 3:** Pharmacokinetic parameters of the FDA-approved oximes. (t_1/2_: half-life excretion time; C_max_: maximum concentration in blood plasma; and T_max_: time to reach C_max_).

Compound	EC50 (mg/L)	t1/2 (h)	Solubility	Cmax (mg/L)	Tmax (h)	Target
Ceftizoxime [113]	*B. fragilis*: 202 *E. cloacae*: 51	5.7–9.4 (rabbits) [107] 1.6–2.57 (people) [114,115,116]	Water (229 mg/L) [117]	34.7	1.5 [118]	*S. aureus:* PBP 2 [119]
Cefpodoxime [120]	*H. influenzae*: 0.04 *M. catarrhalis*: 0.12 *S. pneumoniae*: 0.27 [121]	1.9–2.8	Water (400 µg/mL) [122]	1.0–4.5	1.9–3.1	*E. coli*: Peptidoglycan synthase FtsI [123]
Cefmenoxime [124,125]	ND	1.3–1.5	Water (450 mg/L) [126]	9.07–26.73	0.57–0.77	*E. coli*: Peptidoglycan synthase FtsI [90]
Cefuroxime [127,128]	*K. pneumoniae*: 1.61 [124]	1.2–2.4 [93]	Water (107 mg/L), good in acetone, sparingly soluble in chloroform, ethyl acetate, methanol	4.1–4.8 8.6–9.0	2.0–2.5; 1.8–2.4	*Clostridium perfringens*: PBP 1A [129]

**Scheme 4:**
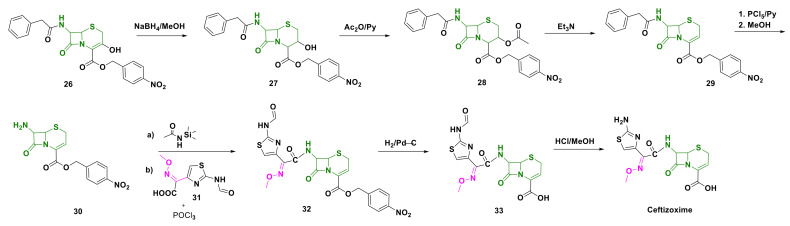
Synthetic scheme of ceftizoxime.

Ceftizoxime is synthesized as shown in Scheme 4 [104]. Synthesis begins with the reduction of 4-nitrobenzylester derivative (**26**), to obtain intermediate **27**. Compound **27** is acylated using acetic anhydride and pyridine to obtain compound **28**, which is then deacetylated to **29**, and further converted to 7-amino intermediate **30** using a mixture of phosphorus pentachloride and pyridine. Compound **30** is first acetylated at the 7-amino position, and subsequently transformed to intermediate **32** by treatment with **31**. The nitro-benzyl ester of compound **32** is further deprotected using palladium over carbon, to obtain intermediate **33** and, ultimately, deformylated to obtain the desired compound, ceftizoxime.

### 4.6. Cefpodoxime

Cefpodoxime is a third-generation antibiotic of the cephalosporin class, active against most Gram-positive and Gram-negative bacteria with good activity against *Hemophilus* spp., *Moraxella* spp., *Klebsiella* spp. and *E. coli (*Figure 12b) [89,130,131]. Usually administered orally, the drug inhibits the cell wall synthesis by interfering with the peptidoglycan synthesis. Cefpodoxime proxetil is de-esterified to its active metabolite-cefpodoxime, by the action of esterase at the intestinal wall. The oral bioavailability is around 50%, after rapid absorption from the gastrointestinal tract within T_max_ of 3 h. The pharmacokinetic studies of cefpodoxime were also carried out and were extensively reviewed [87,89]. Minimum inhibitory concentration (MIC) values were summarized by Todd et al., which report cefpodoxime as a highly effective agent against many species of *Enterobacteriaceae* with MIC values as low as 0.6 µg/mL. However, *Morganella* and *Enterobacter* species, including *P. aeruginosa* and some strains of *M. morganii*, *E. cloacae* and *K. pneumoniae*, were found to be resistant [87]. It was observed that upon oral administration, cefpodoxime accumulated in tonsillar and parotid tissue, lung parenchyma, pleural fluid and inflammatory fluid, and it was recovered unchanged in the urine. Its relatively long t_1/2_ time of excretion (1.9–2.8 h) allowed for less frequent dosing (Table 3) [88,121,132]. Cefpodoxime is effective in curing urinal and respiratory infections, including some skin conditions [133]. It is poorly soluble in water (400 µg/mL) and the recent computational studies showed that it is even less soluble in many other solvents. Cefpodoxime proxetil is an active ingredient of FDA-approved medications, such as discontinued Banan, Vantin and currently prescribed Cefpodoxime Proxetil (Table 2) [134].

**Scheme 5:**
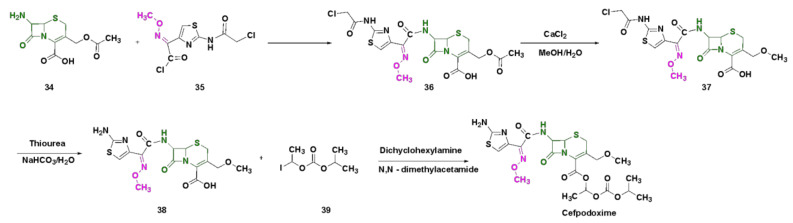
Synthetic scheme of cefpodoxime.

Cefpodoxime is synthesized from 7-ACA, as shown in Scheme 5 [135]. In the first step, 7-ACA (**34**) is treated with the acid chloride of thiazolyl derivative (**35**) to obtain compound **36**. The resulting intermediate (**36**) is treated with aqueous methanol and calcium chloride to obtain the deacetylated intermediate (**37**). In the next step, intermediate **38** is obtained by dechloroacylation using thiourea, which is subsequently treated in the final step with 1-iodoethyl isopropyl carbonate (**39**) to afford the desired compound, cefpodoxime.

### 4.7. Cefmenoxime

Cefmenoxime is a third-generation cephalosporin, predominantly used in the treatment of gynecological and obstetric infections, in addition to nosocomial pneumonia (Figure 12c) [90,136]. Studies revealed the slow development of drug resistance by *E. coli*, *K. preumoniae* and *S. aureus* [91]. On the other hand, the strains of *P. aeruginosa* have a tendency to make cefmenoxime inactive. Gambertoglio et al. evaluated the pharmacokinetics of cefmenoxime in 5 volunteers and 15 patients with renal insufficiency [137]. After a 5 min long intravenous infusion of 10 mg/kg dose, the peak concentration in blood plasma was 95 mg/L, and the cumulative 24 h recovery of the subject compound was 81% for the volunteers; the number was smaller for the patients. Other studies conducted by Polk et al., using 30 min long intravenous infusion of 15 mg/kg dose, were also in line with Gambertoglio’s results [138]. A more detailed study of pharmacokinetics was published by Granneman et al., who determined the excretion and metabolism times, AUC, C_max_ and T_max_ for the different sizes of single intravenous and intramuscular injections of cefmenoxime [125]. Evidently, the C_max_ values were much greater and depended on the dose injected (Table 3). Cefmenoxime is an active ingredient in discontinued Cefmax (Table 2) [139].

**Scheme 6:**
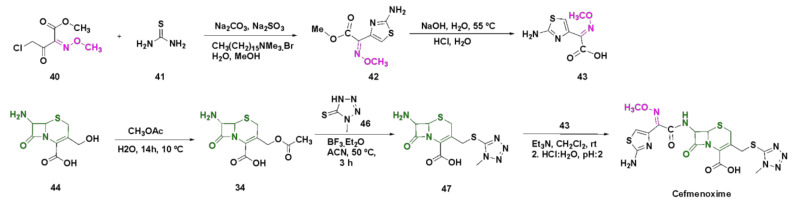
Synthetic scheme of cefmenoxime.

Cefmenoxime can be synthesized, as shown in Scheme 6 [140]. The synthesis of cefmenoxime consists of two parts, which includes the synthesis of two major intermediates: (a) thiazole intermediate (**43**) and (b) 7-amino-cephalosporanic acid derivative (**47**). To begin with, the methoxyimino intermediate (**40**) is treated with thiourea (**41**) to obtain the thiazole intermediate (**42**), which is further hydrolyzed to a 2-amino-thiazole derivative (**43**). The 7-ACA intermediate (**34**) is obtained by the esterification of intermediate (**44**) in an aqueous solution of methyl acetate. The resulting intermediate (**34**) is coupled with the tetrazole intermediate (**46**), in the presence of boron trifluoride to obtain the intermediate **47**, which is subsequently treated with intermediate **43** in the final step to afford the desired compound cefmenoxime.

## 5. Conclusions

The hidden medicinal activity of oximes derivatives found a remarkable place as detoxification agents in the field of OP poisoning, and as ground-breaking antibiotics in the form of cephalosporin derivatives. Currently, six oxime-based drugs were approved by the FDA. Among them, 2-PAM is an FDA-approved oxime, used to restore the activity of AChE in OP poisoning; teboroxime, a myocardial perfusion agent; and the other four being cefuroxime, cefpodoxime, ceftizoxime and cefmenoxime, belonging to the cephalosporins class.

Acetylcholinesterase reactivation has gained a lot of addition, in recent years, with the growing number of suicides resulting from the use of OP agents as insecticides and nerve warfare agents. Although, other oxime-based drugs, such as HI-6, obidoxime and methoxime can also be used for OP poisoning, only 2-PAM has gained the FDA approval for resurrecting the AchE inactivation. With the challenges associated with the so-called “aging” process, toxicity and low blood–brain barrier penetration, there is a rising demand for the development of effective therapies against OP poisoning to overcome these current limitations and the associated side effects. 

On the other hand, alkoxyimino derivatives of cephalosporins feature a higher stability against many known β-lactamases, and are effective against several Gram-positive and Gram-negative bacterial infections. With the readily accessible structure and unique electronic properties of oximes, which make it a hydrogen bond donor and acceptor, a new subclass of cephalosporin anti-bacterial agents has come to light. This novel class of oxime derivatives shows promising anti-bacterial action and great pharmacokinetic parameters over the existing antibiotics.

## Figures and Tables

**Table 1:** Various pharmacokinetic parameters of common oxime based antidotes. (t_1/2_: half-life excretion time; C_max_: maximum concentration in blood plasma; and T_max_: time to reach C_max_). (C_max_ and T_max_ listed for injected concentrations not exceeding 50 mg/kg).

Oxime	EC_50_ (mg/L)	T_max_ (min)	C_max_ (µM)	T_1/2_ min	Status
2-PAM [62]	4.67	10	37–132	37.6–55	Approved
Obidoxime [73,74,75]	57.0	4.5	78	28.4	Approved
HI-6 [37,73,76]	304	5.8	138	25.7	Approved
RS194B [77]	ND	5	112	52	Candidate
K027 [76,78]	229	5	586	60	Candidate
K203 [79]	ND	22	242	101	Candidate

## Data Availability

Data is contained within the article.
